# Synthesis of 5-Hydroxyectoine from Ectoine: Crystal Structure of the Non-Heme Iron(II) and 2-Oxoglutarate-Dependent Dioxygenase EctD

**DOI:** 10.1371/journal.pone.0010647

**Published:** 2010-05-14

**Authors:** Klaus Reuter, Marco Pittelkow, Jan Bursy, Andreas Heine, Tobias Craan, Erhard Bremer

**Affiliations:** 1 Department of Pharmacy, Institute of Pharmaceutical Chemistry, Philipps-University Marburg, Marburg, Germany; 2 Laboratory for Microbiology, Department of Biology, Philipps-University Marburg, Marburg, Germany; Cinvestav, Mexico

## Abstract

As a response to high osmolality, many microorganisms synthesize various types of compatible solutes. These organic osmolytes aid in offsetting the detrimental effects of low water activity on cell physiology. One of these compatible solutes is ectoine. A sub-group of the ectoine producer's enzymatically convert this tetrahydropyrimidine into a hydroxylated derivative, 5-hydroxyectoine. This compound also functions as an effective osmostress protectant and compatible solute but it possesses properties that differ in several aspects from those of ectoine. The enzyme responsible for ectoine hydroxylation (EctD) is a member of the non-heme iron(II)-containing and 2-oxoglutarate-dependent dioxygenases (EC 1.14.11). These enzymes couple the decarboxylation of 2-oxoglutarate with the formation of a high-energy ferryl-oxo intermediate to catalyze the oxidation of the bound organic substrate. We report here the crystal structure of the ectoine hydroxylase EctD from the moderate halophile *Virgibacillus salexigens* in complex with Fe^3+^ at a resolution of 1.85 Å. Like other non-heme iron(II) and 2-oxoglutarate dependent dioxygenases, the core of the EctD structure consists of a double-stranded β-helix forming the main portion of the active-site of the enzyme. The positioning of the iron ligand in the active-site of EctD is mediated by an evolutionarily conserved 2-His-1-carboxylate iron-binding motif. The side chains of the three residues forming this iron-binding site protrude into a deep cavity in the EctD structure that also harbours the 2-oxoglutarate co-substrate-binding site. Database searches revealed a widespread occurrence of EctD-type proteins in members of the *Bacteria* but only in a single representative of the *Archaea*, the marine crenarchaeon *Nitrosopumilus maritimus*. The EctD crystal structure reported here can serve as a template to guide further biochemical and structural studies of this biotechnologically interesting enzyme family.

## Introduction

Environmentally imposed osmotic gradients instigate large water fluxes across the semi-permeable cytoplasmic membrane of microorganisms. As a result, the bacterial cell is threatened by rupture at low osmolality due an undue rise in turgor [1] or by dehydration and a collapse of turgor at high osmolality [2], [3]. At high external osmolality, many microorganisms accumulate a selected group of organic osmolytes, the so-called compatible solutes, to actively counteract the detrimental effects of water efflux on cell physiology [4]. These compounds are fully congruous with metabolism and other cellular functions and can thus be amassed to exceedingly high intracellular levels. As a direct consequence of compatible solute accumulation, the osmotic potential of their cytoplasm is raised. This allows retention of water by the microbial cell, promotes water re-entry, stabilizes turgor and aids the cell in its physiological adjustment to the prevailing osmotic conditions in its environment [2]–[4].

**Figure 1:**
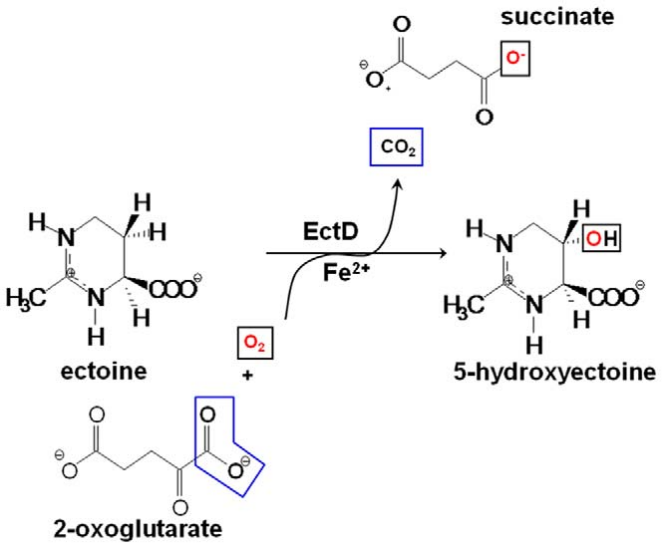
Enzymatic reaction scheme for the EctD-mediated hydroxylation of ectoine.

Ectoine [(*S*)-2-methyl-1,4,5,6-tetrahydropyrimidine-4-carboxylic acid] (Figure 1) is one of the most widely synthesized compatible solutes by microorganisms [4]–[6]. In all ectoine-producing bacteria analyzed so far, ectoine biosynthesis is strongly enhanced under high-osmolality growth conditions. This is largely a consequence of the osmotic induction of the expression of the ectoine biosynthetic genes, *ectABC* [5], [7]–[14]. Three enzymes mediate ectoine biosynthesis from l-aspartate-β-semialdehyde, a central intermediate in amino acid metabolism [15], through the sequential catalytic actions of the l-2,4-diaminobutyrate transaminase (EctB), the l-2,4-diaminobutyrate acetyltransferase (EctA) and the ectoine synthase (EctC) [4]–[6], [11]. A subset of microbial ectoine producers also synthesize a hydroxylated derivative of ectoine, 5-hydroxyectoine [(*S,S*)-2-methyl-5-hydroxy-1,4,5,6-tetrahydropyrimidine-4-carboxylic acid] (Figure 1) [5], [16], [17]. Synthesis of 5-hydroxyectoine depends on the prior production of ectoine. It should be noted in this context that a microbial cell stressed by high salinity cannot gain any advantage in adjusting to high osmolality by enzymatically converting *de novo* synthesized ectoine into 5-hydroxyectoine. This suggests that the physiological function of 5-hydroxyectoine production is not primarily a response to osmotic challenges, but instead is directed towards other types of environmental and cellular stress conditions. Indeed, formation of 5-hydroxyectoine is required in *Chromohalobacter salexigens* to attain full thermo-stress resistance [16]; in addition, *Streptomyces coelicolor* cells challenged by high temperature produce 5-hyroxyectoine in preference to ectoine [7]. Furthermore, the formation of 5-hydroxyectoine is stimulated when either *S. coelicolor* [7], *V. salexigens* [5] or *Marinococcus* M52 [18] cultures become stationary, a growth phase that posesses considerable challenges for microbial cells.

Ectoine and 5-hydroxyectoine are closely related chemically (Figure 1) and both function as effective compatible solutes [18]–[22]. Their cellular accumulation, either *via de novo* synthesis or transport processes [7], [23], [24], can confer protection against salt stress, temperature stress and dehydration [7], [9], [23]–[25]. However, the influence of ectoine and 5-hydroxyectoine on biological macromolecules and cells can differ in several aspects. For instance, ectoine decreases, whereas 5-hydroxyectoine increases the melting temperature of DNA [26], [27]. Furthermore, the protein-stabilizing capacity of these two tetrahydropyrimidines can substantially vary when tested *in vitro* with the same model proteins [19], [28].

Ectoine and 5-hydroxyectoine have attracted considerable biotechnological attention and are currently produced on an industrial scale by high-density fermentation of salt-tolerant bacteria [29]. They have found versatile uses as *in vitro* protein stabilizers, as PCR enhancers, as *in vivo* protein folding catalysts, as whole cell stabilizers and, foremost in skin care products for cosmetics [29], [30]. Medical applications for both ectoine and 5-hydroxyectoine are currently considered [20], [31], [32]. Furthermore, the ectoine biosynthetic genes have been used for the genetic engineering of tobacco plants resistant to salt stress [33].

The ability to form 5-hydroxyectoine is dependent on an evolutionarily conserved ectoine hydroxylase, EctD [5], [7], [16], [17]. The biochemical characterization of this hydroxylase from the moderate halophile *Virgibacillus salexigens* (formerly *Salibacillus salexigens*) [5] and from the soil bacterium *S. coelicolor* [7] has revealed that the EctD enzyme is a member of the non-heme iron(II)-containing and 2-oxoglutarate-dependent dioxygenases superfamily (EC 1.14.11). Members of this enzyme family are widespread in both *Pro*- and *Eukarya* and catalyze a broad spectrum of oxidative reactions including cyclizations, ring fragmentations, C-C bond cleavages, epimerizations, desaturations, halogenations, and hydroxylations of widely varying organic compounds [34]–[36]. This group of enzymes typically couples the decarboxylation of 2-oxoglutarate with the formation of a high-energy ferryl-oxo intermediate that acts as a hydrogen-abstracting species. The formed Fe(IV) = O species is directly responsible for the oxidation of the organic substrate bound by the enzyme. Non-heme iron(II)-containing and 2-oxoglutarate-dependent dioxygenases probably constitute the most versatile group of all oxidizing biological catalysts [37].

Structural studies of these dioxygenases revealed a common protein fold as well as a highly conserved iron-binding motif, the so-called 2-His-1-carboxylate facial triad [34]–[36]. The amino acid sequence of EctD possesses this type of iron-binding motif [5], [7], [16], [17] and EctD catalyzes an enzymatic reaction [5], [7] that is common in non-heme iron(II)-containing and 2-oxoglutarate-dependent dioxygenases [38]. In this reaction, the O_2_-dependent hydroxylation of the substrate ectoine is accompanied by the oxidative decarboxylation of 2-oxoglutarate to form succinate and CO_2_ (Figure 1).

EctD-type proteins are found widely in members of the domain of *Bacteria* [5], [16], but a crystal structure of an ectoine hydroxylase has not yet been reported. Building on the biochemical characterization of the ectoine hydroxylase from *V*. *salexigens* [5], we now report the crystal structure of EctD in complex with Fe^3+^ at a resolution of 1.85 Å. The EctD crystal structure reveals the details of the iron-binding site and also suggests an architectural arrangement for three residues that likely bind and position the co-substrate 2-oxoglutarate with the active site of the EctD enzyme. Our structural data identify the ectoine hydroxylase as a member of the sub-group of PhyH-like enzymes [39], [40] within the non-heme iron(II)-containing and 2-oxoglutarate-dependent dependent dioxygenases superfamily.

## Results and Discussion

### Structure determination of EctD

The X-ray structure of the EctD protein from *V. salexigens* was determined by multi-wavelength anomalous diffraction (MAD) using selenium in selenomethionine as the anomalous scatterer. Crystals of both selenomethionyl-EctD and natural EctD typically showed a mosaicity of more than 2° so that among many obtained crystals only a very few proved to be suitable for X-ray analysis. Since the lowest mosaicity and best resolution was attained from a selenomethionyl-EctD crystal, we used a dataset from this crystal recorded at a remote wavelength for refinement. The crystal structure of EctD was refined at a resolution of 1.85 Å with an *R*_cryst_ of 19.3% and an *R*_free_ of 22.8% (for crystallographic details see Tables S1 and S2). The crystal structure revealed one protein monomer per asymmetric unit, which represents the functional unity of the *V. salexigens* EctD enzyme as previously shown by size exclusion chromatography [5]. One molecule of the *V. salexigens* EctD protein originally consists of 300 amino acids, resulting in a molecular mass of 34.4 kDa. The ectoine hydroxylase from *V. salexigens* used for crystallization was produced in *Escherichia coli* by recombinant DNA techniques and modified with a carboxy-terminal *Strep*-affinity tag for protein purification. However, the *Strep*-tag peptide as well as the carboxy-terminal four amino-acid residues (297 to 300) of the authentic EctD protein were disordered in the crystal and thus excluded from the model of the EctD structure. In addition, a putative loop region extending from amino acid residues 195 to 211 was omitted from the model since no electron density could be assigned to this segment of the EctD protein. The amino-terminal (seleno-) methionine of EctD, although ill-defined in the electron density, was included in the model since the position of its selenium atom was unambiguously identified during the MAD experiment. It represents residue 1 in our numbering system. In addition to 197 water molecules, the refined structure contains one Fe^3+^ ion as well as four non-specifically bound sulfates and two glycerol molecules that originate from the reservoir and cryo-solutions, respectively.

### Overall three-dimensional architecture of EctD

**Figure 2:**
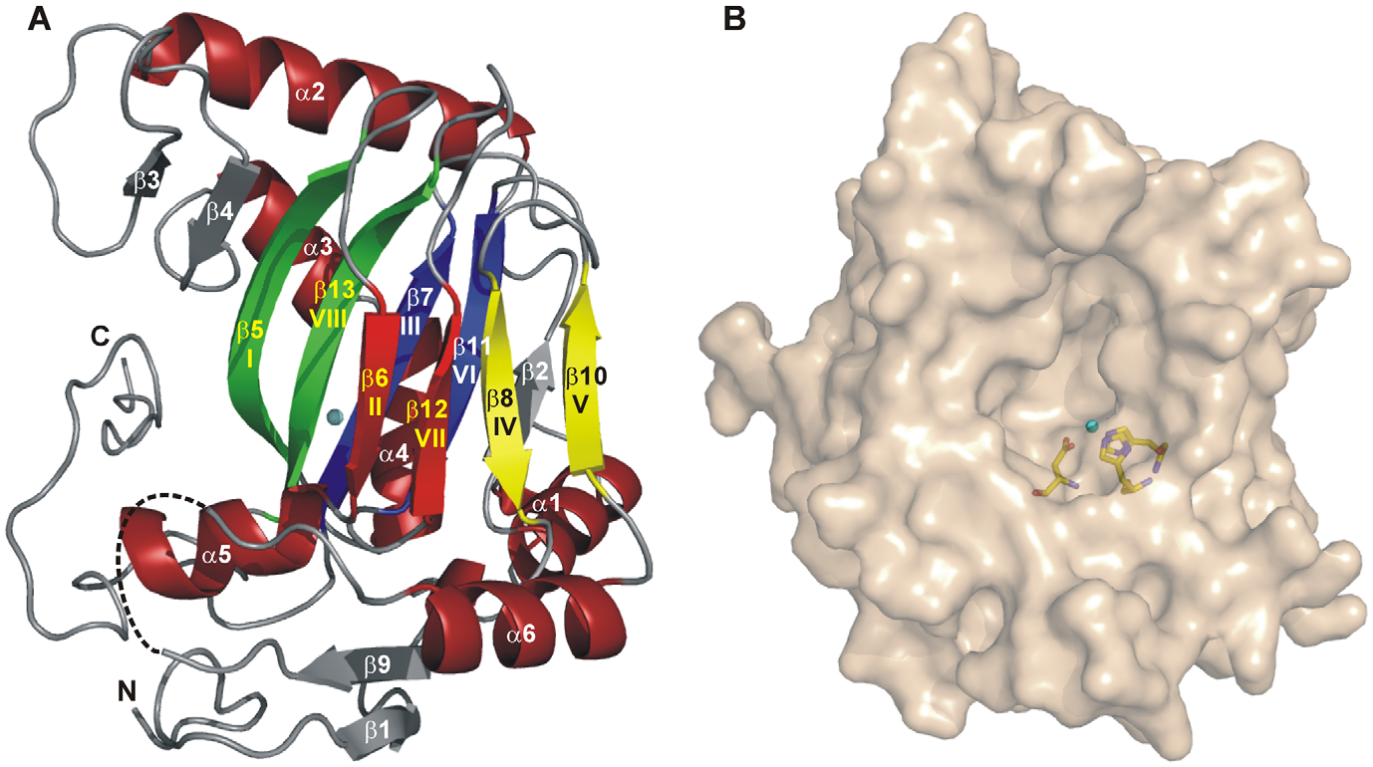
Ribbon and surface representation of the EctD ectoine hydroxylase. (A) The successive segments of the double-stranded β-helix (DSBH) are coloured according to the scheme of Branden and Tooze [75]. The Fe^3+^ ion bound by EctD is shown as a blue sphere. A dashed line indicates a disordered loop region connecting the DSBH β-strands IV and V. (B) The surface of the EctD protein is represented and the side-chains of the iron-coordinating residues His-146, Asp-148 and Asp-248 are shown as sticks. The iron ion bound by the crystallized EctD protein is shown as a blue sphere.

As commonly observed for members of the non-heme iron(II)-containing and 2-oxoglutarate-dependent dioxygenases, the EctD structure consists of a double-stranded β-helix (DSBH) core decorated with and stabilized by a number of α-helices (Figure 2A). The DSBH, also referred to in literature as the jelly-roll fold, is formed by two four-stranded anti-parallel β-sheets which are arranged in the form of a β-sandwich. It is composed of strands β-5, β-6, β-7, β-8, β-10, β-11, β-12, and β-13 (Figure 2A), hereafter referred to as β-I to β-VIII for consistency in nomenclature. As in other non-heme iron(II)-containing and 2-oxoglutarate-dependent dioxygenases, β-II, β-VII, β-IV, and β-V form the distorted minor sheet, which is so called because of its shorter strand lengths, while the major sheet is formed by β-I, β-VIII, β-III, and β-VI. In EctD, the major sheet is extended on both sides by the anti-parallel strands β-2, β-3, and β-4 (Figure 2A).

**Figure 3:**
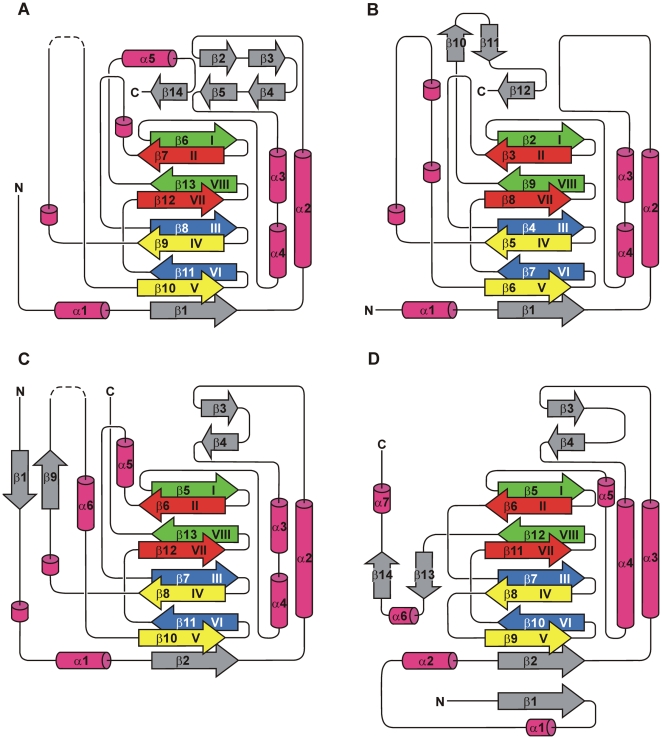
Topology diagrams of PhyH-family enzymes PhyH, SyrB2 and EctD and of the DAOCS enzyme. (A) PhyH (PDB code: 2A1X), (B) SyrB2 (PDB code: 2FCU) and (C) EctD (PDB code: 3EMR). (D) Topology diagram of DAOCS (PDB code: 1DCS), a representative of the family of the IPNS/DAOCS-like enzymes. Arrows represent β-strands, while α-helices are shown as cylinders. 3_10_-helices are also shown as cylinders, but not numbered. The β-strands forming the DSBH-motif are coloured according to the scheme of Branden and Tooze [75]. In the diagrams of EctD and PhyH, the disordered putative “lid” region within the segment connecting DSBH β-strands IV and V is shown by dashed lines. For the sake of clarity and comparability, DSBH β-strand II is included in the topology diagram of PhyH, although this strand is largely disordered and invisible in the corresponding crystal structure [40].

A structural alignment search using the DALI server [41] and the EctD structure as search template revealed that most non-heme iron(II)-containing and 2-oxoglutarate-dependent dioxygenases and related enzymes are structurally similar to EctD (“Z-scores” » 2). Two of these showed a particularly high structural similarity to EctD: the human phytanoyl-CoA 2-hydroxylase PhyH [40] and the halogenase SyrB2 from *Pseudomonas syringae* [42] (Z-scores of 22.5 or 18.2, respectively). Remarkably, the specific substrates of these two enzymes are covalently tethered as thioesters to a phosphopantetheinyl group of CoA (in case of PhyH) or of a peptidyl carrier protein domain (in case of SyrB2), whereas the substrate of EctD is freely diffusible. In the “Structural Classification of Proteins” (SCOP) database [39], PhyH and SyrB2 are combined into the family of PhyH-like enzymes. Our data on the crystal structure of EctD now group this ectoine hydroxylases into the PhyH family as well. In the crystal structures of EctD, PhyH, and SyrB2, an extended region containing four α-helices packed against the major sheet of the DSBH motif precede the amino terminus of the DSBH. This amino-terminal section is highly reminiscent of that present in enzymes of the isopenicillin-*N*-synthase IPNS/deacetoxycephalosporin C synthase (DAOCS)-like family [43], [44]. However, in the members of this family, the helices α-3 and α-4 present in EctD, PhyH, and SyrB2 are fused to one long helix (Figure 3). The major difference between the enzymes of the IPNS/DAOCS family and that of the PhyH family is the way in which β-strands IV and V of the DSBH structural motif are linked with each other. In the IPNS/DAOCS family these strands are connected by a short loop in the structures of the EctD, PhyH, and SyrB2 proteins, but this loop is extended and most likely forms part of the ligand-binding pocket in all three enzymes [40], [42].

When the crystal structures of EctD, PhyH, and SyrB2 are compared, the largest structural differences in these three enzymes occur at their amino and carboxy termini. There are also differences in the region connecting helices α-2 and α-3 (Figure 3). In EctD and PhyH, this region harbours two anti-parallel β-strands that extend the major DSBH sheet by adjoining the carboxy-terminal part of β-strand I. Such anti-parallel β-strands are missing in the corresponding region of SyrB2. Compared to PhyH and SyrB2, the amino terminus of EctD is significantly extended. It contains a β-strand (β-1) that is aligned anti-parallel to β-strand 9 located in the segment connecting DSBH β-strands IV and V. In contrast, both PhyH and SyrB2 are, in comparison to EctD, prolonged at their carboxy termini, which in both enzymes contain a β-strand adjoining anti-parallel to the amino-terminal part of DSBH β-strand I (Figure 3).

### The Fe^2+^ binding site in EctD

**Figure 4:**
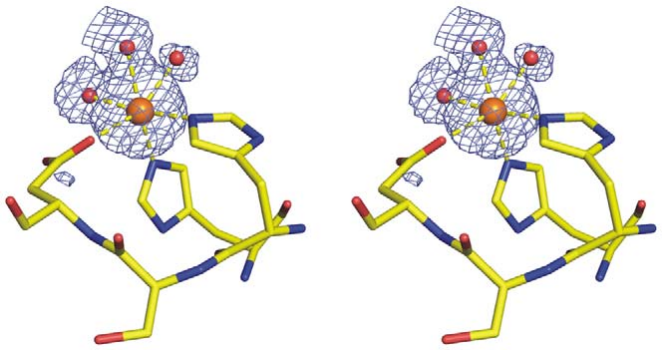
Stereo view of the EctD Fe^2+^-binding site. A co-purified Fe^3+^ (orange sphere) is coordinated by the side chain functional groups of His-146, Asp-148 and Asp-248 of EctD and by three water molecules (shown as red spheres). The |*F_obs_*| – |*F_calc_*| difference electron density (blue mesh) is shown at a sigma level of 3.0 after refinement of the structural model excluding both the Fe^3+^ and its three water ligands.

The enzymatic function of the non-heme iron(II)-containing and 2-oxoglutarate-dependent dioxygenases depends on a highly reactive iron species [35]–[38]. Typically for this enzyme superfamily, the two sheets of the DSBH form the main portion of a deep cavity containing the catalytic Fe^2+^, the binding site for the co-substrate 2-oxoglutarate, and at least part of the substrate-binding site. In most enzymes of this group, the Fe^2+^ ion is coordinated by two histidine imidazoles and the carboxylate of an aspartate or, more rarely, a glutamate residue which together form the so-called 2-His-1-carboxylate facial triad. One of the histidines and the aspartate/glutamate residue are arranged in a conserved His-*X-*Asp/Glu sequence motif [34]–[38]. Although no iron salt had been added to the buffer used for crystallisation of the EctD protein, the experimental electron density clearly indicated a bound metal ion in the active centre of the enzyme that was subsequently refined as an iron ion. It has been previously reported, that the EctD enzyme from *V. salexigens* when heterologously produced in *E. coli* contains a ferric ion [5] and this iron molecule was apparently maintained in the crystallization process of the EctD protein. The metal ion is coordinated by the functional groups of His-146, Asp-148 and His-248 of the EctD protein and three water molecules in an almost perfect octahedral geometry (Figure 4). The temperature- or *B*-factor of an atom within a structural model is a measure of the freedom of movement of this atom. In the structure of EctD, the *B*-factor of the bound metal ion was refined to 23.9 Å^2^ which is below the average *B*-factor of the protein main chain atoms (26.3 Å^2^; Table S2) indicating that the Fe^3+^ is rigidly bound to the protein and excellently defined in the electron density map. The *B*-factors of the water ligands are 31.3 Å^2^, 36.0 Å^2^, and 47.9 Å^2^ (average *B*-factor of all water molecules: 37.2 Å^2^; Table S2). The three residues (His-146, Asp-148, His-248) forming this iron-binding site protrude into a deep cavity in the EctD structure that houses the active site of the enzyme (Figure 2B).

### Residues likely to be involved in 2-oxoglutarate binding by EctD

**Figure 5:**
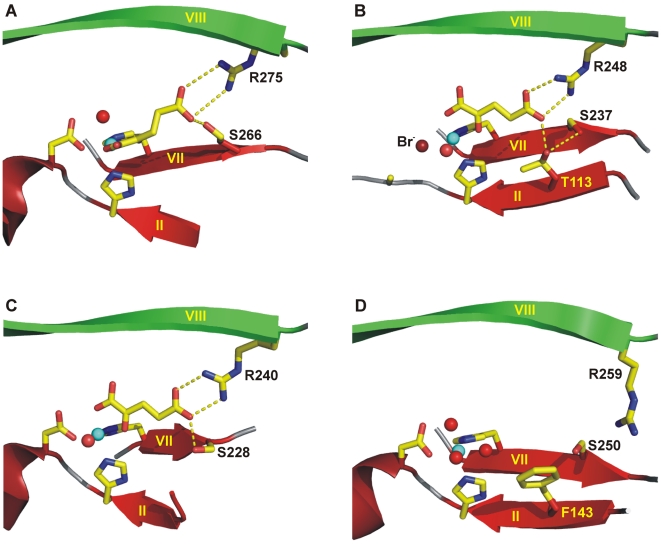
Binding of 2-oxoglutarate by PhyH-like enzymes. (A) PhyH, (B) SyrB2 and (C) PtlH (PDB code: 2RDN) and (D) EctD. The Fe^2+^ ion is shown as a blue sphere, water molecules are shown as red spheres. Side chains of residues involved in 2-oxoglutarate binding or in Fe^2+^ coordination are represented as sticks. Secondary structure elements are indicated as ribbons. The positioning of Phe-143 residue in the EctD structure makes in all likelihood an interaction *via* its aromatic side chain with 2-oxoglutarate. However, the conformation of the Phe-143 side chain shown in this figure must be regarded as tentative as it is poorly defined in the electron density map (in contrast to the Phe-143 main chain atoms). In addition, the actual orientation of the Arg-259 side chain in the EctD crystal structure is not in a position enabling its guanidino function to salt bridge the 5-carboxylate of a bound 2-oxoglutarate. However, a position allowing this interaction can easily be achieved through torsion around rotatable single bonds within the side chain of this Arg residue. It also should be noted that the DSBH β-strand II of PhyH is largely disordered in the crystal structure of this protein. In the EctD structure, the corresponding β-strand also strongly deviates from ideal β-strand geometry.

In all non-heme iron(II)-containing and 2-oxoglutarate-dependent dioxygenases, the co-substrate 2-oxoglutarate is bound at the base of the binding cavity of the enzyme and participates in the coordination of the Fe^2+^ ion *via* its 1-carboxylate and 2-oxo moiety in a bidentate manner. The 5-carboxylate is typically stabilized by a salt bridge formed with the basic group of an arginine or lysine side chain and by at least one hydrogen bond formed to a hydroxyl group of the protein [34]. With few exceptions, the basic residue salt-bridging the 5-carboxylate of 2-oxoglutarate protrudes from the amino-terminus of the DSBH β-strand VIII. An arginine residue is invariantly present in 71 EctD-type proteins compiled by us through database searches (Figure S1) and corresponds to Arg-259 in the *V. salexigens* EctD enzyme (Figure 5D). In members of the IPNS/DAOCS family and a few other non-heme iron(II)-containing and 2-oxoglutarate-dependent dioxygenases, the basic residue and hydroxyl group stabilizing the 5-carboxylate of 2-oxoglutarate stem from an Arg–*X*–Ser sequence motif [34]. This motif is not present in EctD, as residue 261 of the *S. salexigens* enzyme is a non-conserved asparagine residue (Figure S1).

Despite intensive efforts, we were not able to grow EctD crystals in the presence of 2-oxoglutarate. We therefore can only glean information about those residues that might be involved in 2-oxoglutarate binding by comparing the EctD ligand-binding pocket with crystal structures of non-heme iron(II)-containing and 2-oxoglutarate-dependent dioxygenases that actually contain this co-factor. In the PhyH enzyme that is structurally most closely related to EctD, the 2-oxoglutarate-interacting hydroxyl group is provided by Ser-266, positioned two residues away from the Fe^2+^-chelating His-264 residue on DSBH β-strand VII [40] (Figure 5A). The corresponding residue Ser-250 is strictly conserved in 71 EctD-type proteins (Figure S1) and as in PhyH, it is positioned two residues after the invariant Fe^2+^-chelating His-248 (Figure 5D and Figure S1). Furthermore, in the EctD-related SyrB2 crystal structure, two residues after His-235, there is a serine residue (Ser-237) at the corresponding position as well. However, in the SyrB2 halogenase, the hydroxyl group of Ser-237 is not directly involved in 2-oxoglutarate binding. Instead, it forms hydrogen bonds with the hydroxyl group of Thr-113, thereby establishing an additional hydrogen bond to the 5-carboxylate of 2-oxoglutarate (Figure 5B) [42].

Notably, in the recently determined structure of the pentalenolactone biosynthesis enzyme PtlH from *Streptomyces avermitilis*, a serine is also found two residues after the Fe^2+^-chelating His-226 [45]. As in the PhyH structure, Ser-228 of the PtlH enzyme directly hydrogen-bonds the 5-carboxylate of 2-oxoglutarate (Figure 5C). PtlH shows high structural similarity to EctD (DALI Z-score of 16.3) and, according to its overall topology, most likely is a member of the structural family of PhyH-like enzymes as well. With the exception of a prolyl-4-hydroxylase from *Chlamydomonas reinhardtii* [46], none of the remaining non-heme iron(II)-containing and 2-oxoglutarate-dependent dioxygenases with known structure contains a serine at the corresponding position. Therefore, the His-*X*-Ser motif, with the histidine being the distal Fe^2+^-chelating residue and the serine providing an alcohol directly or indirectly stabilizing the 5-carboxylate of the co-substrate 2-oxoglutarate, may represent a typical feature within the family of PhyH-like enzymes.

We noted that the phenyl ring of Phe-143 on DSBH β-strand II protrudes into the presumed binding pocket of the EctD enzyme, where it makes no interactions with any further EctD residue (Figure 5D). Accordingly, the aromatic ring system of this amino acid is freely rotatable and, therefore, ill defined in the electron density. Nevertheless, its positioning suggests that it is able to make, upon binding of 2-oxoglutarate, a stacking interaction with the co-substrate *via* its aromatic side-chain. Consistent with the proposed role of Phe-143 in 2-oxoglutarate binding, replacement of this amino acid by either Ala, Trp or Tyr residues resulted in enzymatically inactive EctD derivatives (M. Pittelkow and E. Bremer; unpublished data). A phenylalanine is not found at an equivalent position in any other non-heme iron(II)-containing and 2-oxoglutarate-dependent dioxygenase of known structure with the exception of the human histone demethylase JMJD2A [47], [48]. Indeed, the corresponding Phe-185 residue in JMJD2A makes *via* its aromatic side chain a Van-der-Waals contact to the bound 2-oxoglutarate or its analogue *N*-oxalylglycine. The histone demethylase JMJD2A shows, however, only moderate overall structural similarity to EctD (DALI Z-score of 5.9).

It should be noted in the context of the discussion of the architecture of the 2-oxoglutarate-binding site, that the actual orientation of the guanidino function of Arg-259, as represented in the EctD crystal structure, is not in a position that would allow Arg-259 to form a salt bridge to 2-oxoglutarate (Figure 5D). This can readily be rationalized in view of the absent 2-oxoglutarate in the EctD active site in the crystallized protein, which will allow flexibility in the positioning of the Arg-259 side-chain. However, an Arg-259 position that allows interaction with 2-oxoglutarate can easily be achieved through torsion around rotatable single bonds within the side chain of this Arg residue.

### A flexible loop in EctD that might be involved in structuring the ligand-binding pocket

The section of the EctD polypeptide chain extending from residues Gly-195 to Leu-211 is disordered in the crystal and consequently not visible in the electron density map. This putative loop region is part of an expanded section of the polypeptide chain connecting DSBH β-strands IV and V of EctD (Figure 2A and Figure 3D). Val-194 and the strictly conserved Gly-212 (Figure S1), two residues that are positioned close to the border of the presumable ligand-binding cavity, flank the disordered loop in the EctD crystal structure. Such an arrangement suggests that this loop might constitute a kind of a flexible “lid” which becomes ordered upon substrate binding.

Notably, in the crystal structure of the EctD-related PhyH protein, the region between residues 223 and 233, which largely corresponds to the disordered loop in EctD, is disordered as well [40]. The PhyH crystal structure was determined in complex with the ligands Fe^2+^ and 2-oxoglutarate but without the PhyH-specific substrate, phytanoyl-CoA. McDonough *et al.* [40] discussed the possibility that the flexible loop present in PhyH might serve to enclose the active site after binding of all reactants by the PhyH enzyme. In the recently determined crystal structure of the non-heme iron(II)-containing and 2-oxoglutarate-dependent asparagine hydroxylase AsnO from *S. coelicolor*, such a lid has been clearly identified [49]. A loop comprising residues 208 to 223 of AsnO is partially disordered in the crystal structure of the apo-form of AsnO but upon ligand-binding, a pronounced structural rearrangement occurs that allows the crystallographic resolution of this flexible segment in AsnO [49]. Strikingly, this mobile lid-structure of AsnO corresponds to the unstructured regions in both the EctD and PhyH crystal structures. As discussed for PhyH [40], Strieker *et al*. [49] also suggest that the flexible loop of AsnO serves to shield the reactants from the surrounding solvent during enzyme catalysis. Furthermore, in the AsnO-related VioC L-arginine oxygenase involved in the biosynthesis of the tuberactinomycin antibiotic viomycin from *Streptomyces vinaceus*, a structurally flexible lid-like region that shields the active site upon substrate binding has been identified as well [50]. In view of the available data on the lid-regions in the PhyH, AsnO, and VioC crystal structures, it is tempting to speculate, that a similar “lid-movement” mechanism might also operate during the catalytic cycle of the EctD-mediated hydroxylation of ectoine.

### A structural perspective on the signature sequence motif of EctD–type ectoine hydroxylases

Based on an alignment of a restricted number of EctD-related proteins, Bursy *et al.* [5] have previously noted a conserved segment of nine amino acid residues (-W^145^HSDFETWH^153^-) and suggested that it might serve as a signature-sequence motif for *bona fide* ectoine hydroxylases. Our updated data base searches for proteins related to the *V. salexigens* ectoine hydroxylase extended this signature sequence motif to F^143^-*X*-WHSDFETWH-*X*-EDG-M/L-P^159^. This motif is invariably present in the amino acid sequence of each of the 71 EctD-type proteins compiled by us, and it stands out as the most conserved part of the ectoine hydroxylase protein sub-family of the non-heme iron(II)-containing and 2-oxoglutarate-dependent dioxygenases (Figure S1). The EctD signature sequence motif comprises two (His-146 and Asp-148) of the three residues forming the iron-binding pocket in the EctD enzyme (Figure 4) and one of the residues (Phe-143) that in all likelihood is involved in 2-oxoglutarate binding (Figure 5D).

**Figure 6:**
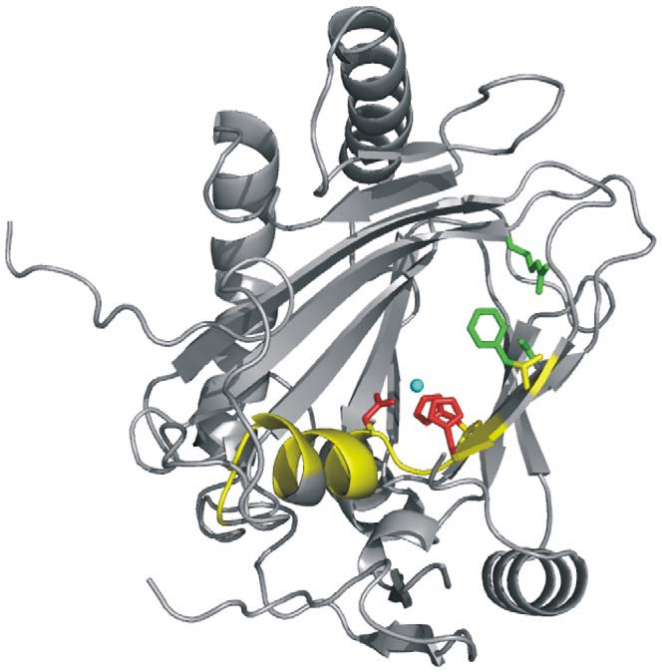
The ectoine hydroxylase signature sequence motif represented in the context of the EctD crystal structure. The *V. salexigens* EctD structure is shown in ribbon representation with those residues constituting the EctD signature motif [F^143^-*X*-WHSDFETWH-*X*-EDG-M/L-P^159^] highlighted in yellow. This string of amino acids is invariantly present in 71 compiled EctD-type proteins (Figure S1). The side chains of the Fe^2+^-chelating amino acids (His-146, Asp-148, His-248) are shown as red sticks and those forming the putative 2-oxoglutarate-binding site (Phe-143, Ser-250, Arg-259) are represented by green sticks. The iron ligand (Fe^3+^) present in the EctD crystal structure is represented as blue sphere.

It is now possible to inspect the F^143^-*X*-WHSDFETWH-*X*-EDG-M/L-P^159^ motif within the framework of a crystal structure of a biochemically characterized ectoine hydroxylase. Most of the residues forming the ectoine hydroxylase signature-sequence motif are structurally organized as an α-helix (α-helix 5; Figure 3D and Figure 6). This helix consists of nine amino acids (Asp-148 to Asp-156), all of which, except Val-154, are strictly conserved in EctD-type proteins (Figure S1). This is in contrast to the other five α-helices present in the EctD structure (Figure 3D), none of which contains a single invariant amino acid (Figure S1). Along with the carboxy-terminal loop of EctD and β-strands 3 and 4, α-helix 5 expands the ligand-binding cavity, whose core is formed by the DSBH motif (Figure 2A and Figure 6). In analogy to what is observed in crystal structures of EctD-related non-heme iron(II)-containing and 2-oxoglutarate-dependent dioxygenases with bound substrates, this expansion is likely to form an important part of the ectoine-binding site. This assumption is strengthened when one considers the spatial proximity within the EctD structure of α-helix 5 to residues Val-194 and Gly-212, two residues that flank the putative “lid” region of the EctD enzyme.

Despite intensive efforts, we have not been able to obtain EctD crystals with either the substrate ectoine or the product 5-hydroxyectoine. We can thus currently not tell how or where the ectoine ligand is bound within the presumed EctD active site. Recently, the crystal structures of ectoine/5-hydroxyectoine ligand-binding proteins from an ABC and two TRAP transporters have been reported. The binding of both compatible solutes by EhuB from *Sinorhizobium meliloti* [51], TeaA from *Halomonas elongata* [52], and UehA from *Silicibacter pomeroyi* [53] relies on cation-π interactions between the positive charge of both ectoine and 5-hydroxyectoine (Figure 1) and aromatic side chains of the ligand-binding proteins and on directed protein-ligand interactions *via* salt-bridges and hydrogen bonds. In view of this information, we inspected the likely active centre of the EctD enzyme (Figure 2B and Figure 6) for an arrangement of amino acid side chains similar to that found within the ectoine/5-hydroxyectoine-binding pockets of EhuB, TeaA and UehA. However, the presumed active site of the EctD enzyme apparently does not contain an ectoine/5-hydroxyectoine-binding site that closely matches that is present in these ligand-binding proteins.

### EctD-type proteins within the context of microbial genomes and microbial physiology

The structural gene (*ectD*) for the ectoine hydroxylase is either part of the ectoine biosynthetic gene cluster or it is encoded separately from *ectABC* operon somewhere else in microbial genomes [5], [15]. Using the amino acid sequence of the ectoine synthase (EctC) as a diagnostic tool to identify potential ectoine producers *via* data-base searches, we identified 197 microbial species as putative ectoine producers. Of these, 67 species were also found to contain EctD-type ectoine hydroxylases. Four microbial genomes carried two copies of *ectD* genes.

The 71 compiled EctD-type proteins exhibit a degree of amino acid sequence identity to the EctD protein from *V. salexigens* that ranges from 55 to 41%. These proteins comprise a similar number of amino acid residues and all align with each other without major gaps (Figure S1). Only a sub-group of these EctD-type proteins are currently functionally annotated in the databases as ectoine hydroxylases. Many EctD-type proteins are referred to as either proline- or phytanoyl-CoA-hydroxylases, or are non-specifically referred to as probable hydroxylases/dioxygenases. Proline-hydroxylases are members of the non-heme iron(II)-containing and 2-oxoglutarate-dependent dioxygenase superfamily (EC 1.14.11) and carry out an enzymatic reaction similar to that catalyzed by the EctD ectoine hydroxylase [54], [55], but the amino acid sequence of this type of enzymes can readily be distinguished from those of ectoine hydroxylases in BLAST searches. Our finding that EctD is structurally closely related to the human phytanoyl-CoA-hydroxylase PhyH (Figure 3A), probably explains the annotation of many EctD-type hydroxylases in the databases as putative microbial phytanoyl-CoA-hydroxylases.

Microbial genomes usually contain only one copy of the *ectD* gene but we found four exceptions: *Rhodococcus sp.* RHA1, *Rhodococcus opacus* B4, *Marinobacter aquaeolei* VT8 and *C. salexigens* each encode two EctD-type proteins (Figure S1). In the case of *C. salexigens*, the contributions of the two EctD-type proteins (referred to as EctD and EctE, respectively) for the synthesis of 5-hydroxyectoine have already been investigated through gene disruption experiments. Only the EctD protein significantly contributes to 5-hydroxyectoine production, despite the fact that the amino acid sequence of EctD and EctE of *C. salexigens* are 50% identical [16].

Ectoine and 5-hydroxyectoine were so far considered as compatible solutes exclusively synthesised by members of the domain of the *Bacteria* [4]. However, our database searches revealed an EctD-type protein encoded in the genome of the archaeon *Nitrosopumilus maritimus SCM1* (NCBI accession number NC_010085). The *ectD* gene of this marine group 1 *Crenarchaeon* [56] is part of a gene cluster (*ectABCD*) that encodes the three enzymes required for ectoine biosynthesis as well. There is considerable evidence for extensive lateral gene transfer between members of the domains of the *Bacteria* and the *Archaea* [57], [58]. It is thus tempting to speculate that *N. maritimus SCM1* acquired the ectoine and 5-hydroxyectoine biosynthetic genes *via* lateral gene-transfer from a member of the *Bacteria* that shares the marine habitat with this archaeon. The presence of the ectoine/5-hydroxyectoine gene cluster in *N. maritimus SCM1* was recently also noted by Lo *et al*. [15] in connection with a cohesion group approach for the evolutionary analysis of microbial aspartokinases, enzymes that are crucial for the production of l-aspartate-β-semialdehyde, the precursor for the biosynthesis of ectoine [15], [59].

EctD-possessing microorganisms are quite diverse with respect to their taxonomic affiliation, physiology, lifestyles and habitats (Figure S1). There are well-known pathogens among this group of bacteria such as *Norcadia farcina*, *Mycobacterium abscessus* and various species of *Bordetella*. But there are also representatives of biotechnologically important microorganisms such as *S. coelicolor* and *Streptomyces griseus,* which are both employed for antibiotic production. Represented are also microorganisms that live in extreme habitats with respect to growth temperature (e.g. the thermophilic *Geobacillus sp* Y412MC10 and the psychrophilic *Sphingopyxis alaskensis* RB2256) and pH tolerance (e.g. *Acidiphilum cryptum* JF5 and *Alkalilimnicola ehrlichei* MLHE-1). A substantial number of *ectD*-possessing microorganisms inhabit high-salinity environments or marine habitats. One representative of this group is the cosmopolitan hydrocarbonoclastic marine bacterium *Alcanivorax borkumensis* SK2, whose ability to synthesize ectoine and 5-hydroxyectoine is key for its ability to colonize high salinity-habitats [60]. It will be interesting to see in future studies how the EctD-mediated synthesis of 5-hydroxyectoine contributes to the specific adaptation of microorganisms to their varied ecological niches.

## Materials and Methods

### Chemicals

Ectoine and 5-hydroxyectoine either were purchased from Biomol (Hamburg, Germany) or were a kind gift from Dr. T. Schwarz (Bitop AG, Witten, Germany). 2-oxoglutarate (disodium salt) was obtained from Sigma-Aldrich (St. Louis, MO, USA). Anhydrotetracycline-hydrochloride, desthiobiotin, and *Strep-*Tactin Superflow chromatography material were purchased from IBA (Göttingen, Germany).

### Culture conditions for bacterial strains

*E. coli* strains were grown aerobically in Luria-Bertani (LB) rich medium and were propagated on LB-agar plates at 37°C [61]. Ampicillin (100 µg mL^−1^) was added to liquid and solid media to select for the presence of the *ectD* ^+^ plasmid pBJ10 [5] in the *E. coli* strain DH5α (Invitrogen, Karlsruhe, Germany). Overproduction of the EctD protein was performed in minimal medium A [61] with 0,5% (w/v) glucose as the carbon source, 0.5% (w/v) casaminoacids, 1 mmol L^−1^ MgSO_4_ and 3 µmol L^−1^ thiamine.

### Overproduction and purification of the recombinant EctD protein in *E. coli*

Plasmid pJB10 is a derivative of the expression vector pASK-IBA3 (IBA, Göttingen, Germany) and carries the *ectD* ^+^ gene from *V. salexigens* (DSM 11483^T^) under the transcriptional control of the anhydrotetracycline-inducible *tet*-promotor present on pASK-IBA3. The *ectD* coding region is fused at its 3′-end to a DNA segment that encodes a *Step*-tag-II affinity peptide. Expression of the recombinant *V. salexigens ectD* gene in *E. coli* DH5α (pBJ10) was triggered by the addition of anhydrotetracyline (final concentration: 0.2 µg mL^−1^) to mid-log-phase-cultures (OD_578_ of 0.7) propagated in minimal medium A. Growth of the cells was continued for two hours after the addition of the inducer to allow EctD*-Strep*-tag-II protein production, and the cells were subsequently harvested by centrifugation (4°C, 10 min at 5 000×g). EctD*-Strep*-tag-II production was carried out in batch-cultures (6×1 liter minimal medium A). The recombinant EctD*-Strep*-tag-II protein was purified from cleared cell lysates of the *E. coli* DH5α (pJB10) producer cells by affinity chromatography on a *Strep-*Tactin Superflow column as detailed by Bursy *et al.* [5]. In general, approximately 8 mg of purified EctD*-Strep*-tag-II protein were obtained per liter of *E. coli* DH5α (pJB10) culture. The purified EctD*-Strep*-tag-II protein was shock-frozen in liquid nitrogen and stored at −80°C for subsequent enzyme assays [5] and crystallization trials.

### Crystallization of the EctD protein from *V. salexigens*

The EctD*-Strep*-tag-II protein was crystallized at 293 K by the hanging-drop vapour diffusion method using VDX™ plates (Hampton Research, USA). For an initial screen, the 98 solutions of the Crystal Screen and Crystal Screen II kit from Hampton Research (USA) were used. 1.5 µL of protein solution (10 g L^−1^ EctD protein in 10 mmol L^−1^ TES, pH 7.5; 80 mmol L^−1^ NaCl; 2 mmol L^−1^ DTT) were mixed with 1.5 µL reservoir solution and equilibrated against 0.5 mL of reservoir solution. A large number of very small crystals appeared overnight in 2.0 mol L^−1^ (NH_4_)_2_SO_4_ buffered with 0.1 mol L^−1^ sodium acetate (pH 4.6); Crystal Screen #47). Optimization of this condition using the Additive Screen kit from Hampton Research (USA) led to a reservoir solution containing 1.0 mol L^−1^ (NH_4_)_2_SO_4_, 0.1 mol L^−1^ NaF, 2 mmol L^−1^ TCEP, 0.02% (*w/v*) sodium azide buffered with 0.1 mol L^−1^ sodium acetate at pH 5.0. When 2 µL of protein solution were mixed with 3 µL of the refined reservoir solution, large crystals were obtained after five days. The crystals were of bipyramidal shape with a hexagonal base and were approximately 0.8 mm in length and 0.5 mm in width. Only a small fraction of the obtained crystals showed good diffraction behaviour. Omitting NaF from the reservoir solution inevitably resulted in morphologically imperfect crystals exhibiting an extremely high mosaicity. For a multiwavelength anomalous diffraction (MAD) experiment, selenomethionine (SeMet)-substituted EctD was produced according to the metabolic inhibition protocol established by Van Duyne *et al.* [62] with slight modifications. The presence of the SeMet in the protein was confirmed by matrix-assisted laser desorption/ionisation (MALDI-TOF) mass spectrometry. Purification was identical to sulfurmethionine EctD. For crystallization of SeMet-labelled EctD the concentration of (NH_4_)_2_SO_4_ in the reservoir solution had to be decreased to 0.9 mol L^−1^.

### Data collection, processing, and structure determination by means of multiwavelength anomalous diffraction (MAD)

For data collection at 100 K, crystals were cryopreserved in a reservoir solution with an increased (NH_4_)_2_SO_4_ concentration (1.4 mol L^−1^) plus 30% (*v/v*) glycerol. The crystals belonged to the hexagonal space group P6_5_22 with unit cell dimensions of *a* = *b* = 102.7 Å, *c* = 158.8 Å. Calculation of a Matthews coefficient of 3.7 Å^3^/Da implied one protein monomer per asymmetric unit, yielding a solvent content of 66.9%. The crystal structure of EctD was determined by MAD using selenium of SeMet incorporated into the EctD protein as anomalous scatterer [63]. The MAD experiment was performed at the Proteine Structure Factory (PSF) beamline 14.2 at the Berliner Elektronenring-Speicherring für Synchrotronstrahlung (BESSY; Berlin, Germany) equipped with a MAR-165CCD detector. One crystal of selenomethionyl EctD was used to collect a MAD data set at three different wavelengths at 2.1 Å resolution each (1° oscillation steps at a crystal to detector distance of 150 mm). Subsequently, data of 1.85 Å resolution were collected from a further crystal of selenomethionyl EctD (0.5° oscillation steps at a crystal to detector distance of 135 mm) at the remote wavelength. This data set was used as the “native” data set and for refinement of the EctD crystal structure. All data were processed and scaled with the programs DENZO and SCALEPACK implemented in the HKL2000 package [64]. For MAD data the option ‘NO MERGE ORIGINAL INDEX’ was used to allow local scaling. Data collection statistics are summarized in Table S1. The data quality was examined with SHELXC as implemented in HKL2MAP [65]. The “native” data set was used as a reference for the calculation of the correlation coefficient. The anomalous signal/noise (>1.5) and the correlation coefficient (>30) between data sets were significant up to a resolution of 2.2 Å. The selenium substructure was determined with the program SHELXD [66]. Five of the seven selenium atoms present in the SeMet-labelled EctD protein were found. Among these was the selenium atom of the *N*-terminal methionine of EctD, which was only poorly defined both in the experimental and refined electron density map. In addition, a complexed Fe^3+^ ion showing a week anomalous signal at the wavelengths used for the MAD experiment was identified. The correlation coefficient for the best solution was 71.0 and the *R*-factor was 21.1%. Reference phases from the selenium positions were calculated and improved by density modification using SHELXE [67]. Phase extension was performed with the “native” dataset to 1.85 Å resolution. The correct enantiomorph of the Patterson function returned a single solution with a final contrast of 1.00 and a connectivity of 0.95 (inverted site ≡ P6_1_22: contrast: 0.07, connectivity: 0.73).

### Model building and crystallographic refinement

The automatic model building program ARP/wARP was applied for automatic tracing of the electron density and model building [68]. After 200 refinement cycles, this procedure provided an initial model consisting of 276 out of 300 amino acid residues (not taking into account the carboxy-terminal *Strep*-affinity tag) with a connectivity index of 0.99. Residues 195 to 212 as well as the six *C*-terminal residues 295 to 300 were missing in this first model. A Fe^3+^ ion that was clearly visible in the experimental electron density was manually introduced. Initially, simulated annealing and B-factor refinement was performed in CNS [69]. After some cycles of refinement, the refined electron density allowed building of residues 212, 295 and 296 using the program O [70]. In addition, the refined electron density clearly revealed two alternative side chain conformations for residues SeMet-63, Ser-73, Asp-101, Asp-124 and Ile-229. The alternative side chain conformations, two glycerol molecules and four sulfate ions that were visible in the electron density, were built in O. Further refinement of the model was done using the program SHELXL [71] until the *R*_free_ no longer decreased (see Table S2 for refinement and model statistics). Since no or only poor electron density could be assigned to the side chains of Glu-2, Leu-4, Gln-9, Asn-10, Asn-11, Gln-12, Lys-14, Lys-17, Glu-38, Glu-85, Glu-87, Lys-141, Phe-143, Lys-292, Gln-293 and Val-295, these residues were modelled as alanines. The ϕ,ψ-angle combination of Glu-87 whose main chain portion was excellently defined in the electron density map is in the forbidden area of the Ramachandran plot. As analyzed by means of the program PROMOTIF [72], it is present in a type IV beta turn motif.

### Protein Data Bank accession number

The coordinates of the EctD crystal structure in complex with Fe^3+^ were deposited with the Brookhaven Protein Data Bank under accession number 3EMR.

### Figure preparation

Structural figures were prepared using Pymol (http://www.pymol.org).

### Database searches and computer analysis of protein sequences

Proteins that are homologous either to the EctC or EctD proteins from *V. salexigens* were searched *via* the Web-server of the DOI Joint Genome Institute (http://www.jgi.doe.gov/) or that of the National Center for Biotechnology Information institute (http://www.ncbi.nlm.nih.gov/) using the Blast algorithm [73]. The genome context of finished and unfinished microbial genomes in the vicinity of the *ectD* gene was analyzed using the gene neighbourhood tool (http://img.jgi.doe.gov/cgi-bin/pub/main.cgi) provided by the JGI Web-server. Sequence alignments of EctD-type proteins were performed using ClustalW [74] as implemented in the Vector NTI software package (Invitrogen, Karlsruhe, Germany).

## Supporting Information

Figure S1 The *Virgibacillus salexigens* EctD amino acid sequence (accession number: AAY29689) was used as a query sequence in BLAST searches using the web tools of the National Center for Biotechnology Information (NCBI; http://www.ncbi.nlm.nih.gov/) or the Joint Genome Institute (JGI; http://www.jgi.doe.gov/). The retrieved amino acid sequences were then aligned using the ClustalW algorithm as implemented in the Vector NTI DNA analysis software. The position of those amino acids that are involved either in iron or in 2-oxoglutarate binding by the *V. salexigens* EctD protein are labelled by red and green boxes, respectively. The numbering of these residues is according to the amino acid sequence of the *V. salexigens* EctD protein.
(0.36 MB PDF)

Table S1 X-ray data collection statistics for the EctD protein.
(0.03 MB DOC)

Table S2 Refinement and model statistics for the EctD crystal structure (PDB code: 3EMR).
(0.03 MB DOC)
