# Supplementary material for: Synthesis of 5-Hydroxyectoine from Ectoine: Crystal Structure of the Non-Heme Iron(II) and 2-Oxoglutarate-Dependent Dioxygenase EctD
Source: PLoS One. 2010 May 14;5(5):e10647. doi: 10.1371/journal.pone.0010647 (PMC2871039; doi:10.1371/journal.pone.0010647)
Supplement: Figure S1 — The Virgibacillus salexigens EctD amino acid sequence (accession number: AAY29689) was used as a query sequence in BLAST searches using the web tools of the National Center for Biotechnology Information (NCBI; http://www.ncbi.nlm.nih.gov/) or the Joint Genome Institute (JGI; http://www.jgi.doe.gov/). The retrieved amino acid sequences were then aligned using the ClustalW algorithm as implemented in the Vector NTI DNA analysis software. The position of those amino acids that are involved either in iron or in 2-oxoglutarate binding by the V. salexigens EctD protein are labelled by red and green boxes, respectively. The numbering of these residues is according to the amino acid sequence of the V. salexigens EctD protein. (0.36 MB PDF) [file pone.0010647.s001.pdf]

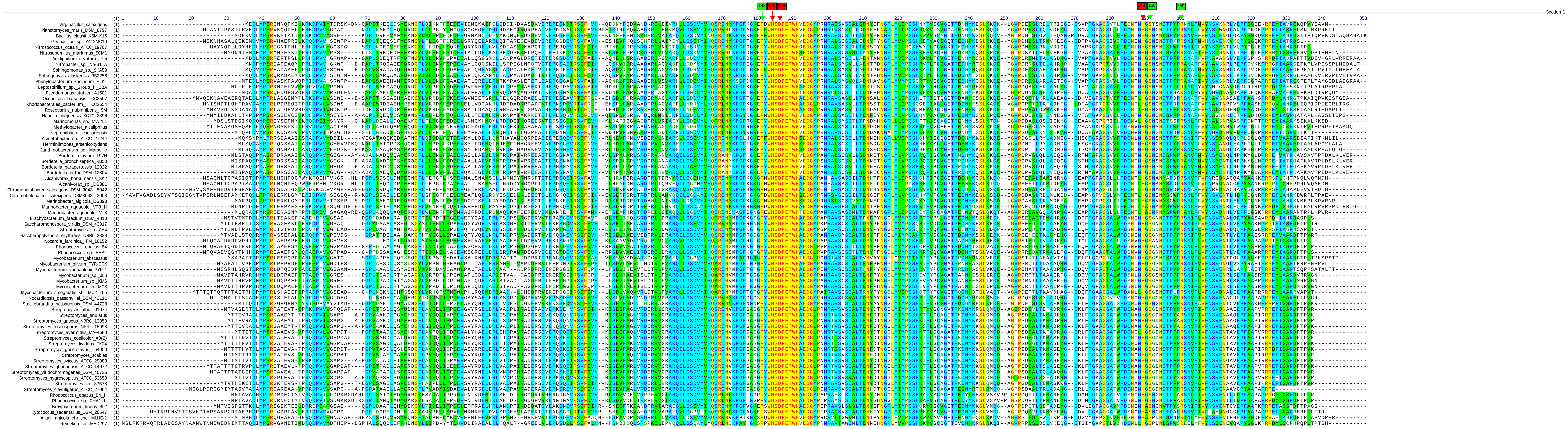
Section 1
