# Supplementary material for: Synthesis of 5-Hydroxyectoine from Ectoine: Crystal Structure of the Non-Heme Iron(II) and 2-Oxoglutarate-Dependent Dioxygenase EctD
Source: PLoS One. 2010 May 14;5(5):e10647. doi: 10.1371/journal.pone.0010647 (PMC2871039; doi:10.1371/journal.pone.0010647)
Supplement: Table S1 — X-ray data collection statistics for the EctD protein. (0.03 MB DOC) [file pone.0010647.s002.doc]

**Table S1.** X-ray data collection statistics for the EctD protein

|  | MAD data | | | "native" |
| --- | --- | --- | --- | --- |
|  | Peak | Inflection | Remote |  |
| Wavelength [Å] | 0.97971 | 0.97997 | 0.90810 | 0.90810 |
| Unit-cell parameters |  | | | |
| *a* = *b* [Å] | 102.67 | 102.70 | 102.71 | 102.79 |
| *c* [Å] | 158.78 | 158.82 | 158.83 | 159.07 |
| a = b [°] | 90 | 90 | 90 | 90 |
| g [°] | 120 | 120 | 120 | 120 |
| Space group | *P*6_5_22 | *P*6_5_22 | *P*6_5_22 | *P*6_5_22 |
| Resolution range [Å] | 50.0 – 2.10 | 50.0 – 2.10 | 50.0 – 2.10 | 50 – 1.85 |
| mosaicity [°] | 0.14 | | | 0.15 |
| Total observations | 452790 | 452723 | 419285 | 629315 |
| Unique observations | 54625 | 54678 | 54777 | 43040 |
| Redundancy | 8.3 | 8.3 | 7.7 | 14.6 |
| Completeness [%] | 99.9 (98.4) | 99.9 (98.0) | 99.9 (98.0) | 99.9 (100.0) |
| *I* / s | 26.4 (4.2) | 28.2 (4.1) | 24.7 (3.6) | 34.1 (5.9) |
| *R*_sym_ [%] | 8.5 (30.9) | 7.8 (31.3) | 8.0 (32.6) | 6.7 (43.7) |
| Numbers in parentheses are for the highest-resolution shell (1.85 Å – 1.88 Å).  , where *I* is the observed intensity and *Ī* is the average intensity for multiple measurements. | | | | |
