# Supplementary material for: Synthesis of 5-Hydroxyectoine from Ectoine: Crystal Structure of the Non-Heme Iron(II) and 2-Oxoglutarate-Dependent Dioxygenase EctD
Source: PLoS One. 2010 May 14;5(5):e10647. doi: 10.1371/journal.pone.0010647 (PMC2871039; doi:10.1371/journal.pone.0010647)
Supplement: Table S2 — Refinement and model statistics for the EctD crystal structure (PDB code: 3EMR). (0.03 MB DOC) [file pone.0010647.s003.doc]

**Table S2.** Refinement and model statistics for the EctD crystal structure (PDB code: 3EMR)

| Resolution range in refinement [Å] | 50 – 1.85 |
| --- | --- |
| *R_cryst_ ^a^* [%] | 19.3 |
| *R_free_^b^* [%] | 22.8 |
| Deviations from ideal geometry  Bond lengths [Å]  Torsion angles [°] | 0,009  2.1 |
| Number of non-*H* atoms  Protein  Fe(III)  SO_4_^2-^  Glycerol  Water molecules | 2223  1  20  12  197 |
| Average B-factors [Å^2^]  Protein  Main chain  Side chain  Glycerol  SO_4_^2-^  Fe^3+^  Water | 28.1  26.3  30.0  42.9  43.5  23.9  37.2 |
| Ramachandran plot [%]  Most favoured  Additionally allowed  Generously allowed  Disallowed | 90.1  9.5  0  0.4 |
| ^a^ $R_{cryst}=\frac{\sum_{hkl} \left\vert F_{obs}-F_{calc}\right\vert}{\sum_{hkl} \left\vert F_{obs}\right\vert}.$ ^b^ *R_free_* was calculated as *R_cryst_* but with 5% of the data excluded from the refinement. | |
